# Respiratory, cardio-metabolic and neurodevelopmental long-term outcomes of moderate to late preterm birth: not just a near term-population. A follow-up study

**DOI:** 10.3389/fmed.2024.1381118

**Published:** 2024-08-20

**Authors:** Patricia Alonso-Lopez, Maria Arroyas, Maite Beato, Sara Ruiz-Gonzalez, Iciar Olabarrieta, Maria Luz Garcia-Garcia

**Affiliations:** ^1^Department of Pediatrics, Hospital Universitario Severo Ochoa, Madrid, Spain; ^2^Instituto de Investigación Sanitaria Puerta de Hierro—Segovia de Arana, Hospital Universitario Puerta de Hierro Majadahonda, Majadahonda, Spain; ^3^Networked Biomedical Research Center for Infectious Diseases (CIBERINFEC), Madrid, Spain; ^4^Traslational Research Network in Pediatric Infectious Diseases (RITIP), Madrid, Spain

**Keywords:** moderate to late preterm, premature birth, asthma, lung function, cardiovascular risk, metabolic risk, developmental disabilities

## Abstract

**Introduction:**

Moderate-to-late preterm infants constitute the majority within the preterm infant population. Most research on preterm infants has focused on very preterm children, often treating moderate-to-late preterm infants as similar to full-term infants. Our objective was to compare clinical, respiratory, cardio-metabolic and neurodevelopmental outcomes in adolescents aged 12–15 years born moderate and late preterm with a control group of the same age born full-term.

**Methods:**

Observational cross-sectional study, comparing moderate-to-late preterm (32–36^+6^ weeks’ gestational age) with full-term adolescents (37–41^+6^ weeks’ gestational age; 75 each group). Perinatal and neonatal history were collected as well as data on respiratory evolution (ISAAC questionnaire for asthma symptoms for adolescents 13–14 years), anthropometric values, learning difficulties, behavioral test (screening questionnaire for high-performance autism spectrum disorder and evaluation test for attention deficit hyperactivity disorder), skin prick test, pulmonary function test, echocardiogram and blood pressure. A blood test with metabolic profile was conducted.

**Results:**

Moderate-to-late preterm adolescents had more current asthma [*p* = 0.008, OR3 (95% CI 1.26–7.14)] and longer duration of combined treatments to control asthma (inhaled corticosteroids and anti-leukotrienes; *p* = 0.048). Forced vital capacity <80% was detected more often in moderate-to-late preterm patients (*p* = 0.013). When assessing right ventricle, moderate-to-late preterm adolescents showed better tricuspid annular plane systolic excursion z-score (*p* = 0.003), shortening fraction (*p* < 0.001) and E/A ratio z-score (*p* = 0.002). Regarding left ventricular assessment, moderate-to-late preterm group had smaller ventricle diastolic diameter (*p* = 0.04) and lower posterior wall z-score values (*p* = 0.037). They also showed a better S’wave z-score (*p* = 0.027), E wave (*p* = 0.005), E/A ratio (*p* = 0.003) and a higher septal myocardial performance index z-score (*p* = 0.025). Moderate-to-late preterm adolescents presented lower weight z-score (*p* = 0.039), body mass index z-score (*p* = 0.013), Waterlow weight index (*p* = 0.006) and higher undernutrition index [*p* = 0.04; OR 1.4 (95% CI 1–1.9)]. Although there were no differences in neurodevelopmental survey or behavioral tests.

**Conclusion:**

Our findings underscore the importance of extended follow-up for this predominant group of premature infants to identify potential respiratory, cardiac and anthropometric issues that may emerge in the future.

## Introduction

Preterm infants account for 10.6% of livebirths (1). Preterm birth, defined as birth before 37 weeks, is a very heterogeneous group. Moderate-to-late preterm (MLP) infants, which are defined as birth between 32 and 36 weeks’ gestation and represents 85% of all preterm births (1, 2).

**Figure 1:**
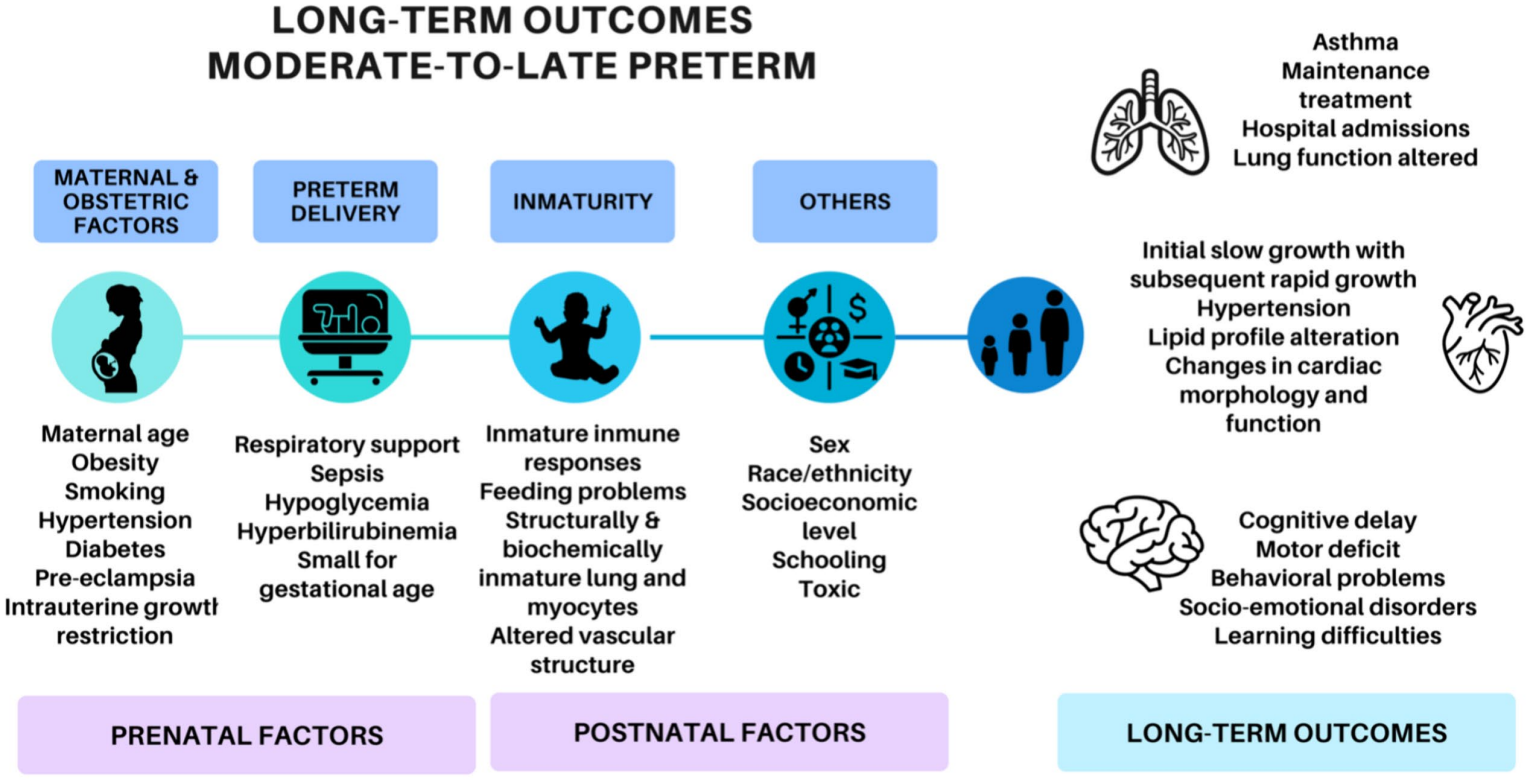
Factors involved in the development of the long-term outcomes of moderate-to-late preterm adolescents.

Previously, MLP infants have been considered “near” to term. However, recent publications (3, 4) report that MLP births have higher rates of morbidity and mortality compared to full-term children, especially in the first year of life. In addition, most recent studies (5, 6) suggest cardiovascular, neurodevelopmental and respiratory adversity in their evolution (Figure 1). These studies have revealed that MLP birth can be associated with poor growth (7, 8), increased blood pressure (4, 9), dyslipidemia or insulin resistance (10, 11), but outcomes in the adolescence have been inconsistent.

Lung development is also affected in MLP births. Compared with term, MLP children suffer more bronchiolitis, requiring hospitalization, and asthma especially in childhood (12–14). As the age of infants born MLP increases, a lower prevalence of asthma has been reported (15). It has been described in MLP infants a similar forced vital capacity (FVC) and forced expiratory volume in 1 s (FEV_1_), but lower mean forced expiratory flow between 25% and 75% of FVC (FEF_25–75_) than in term infants (15, 16). However, the impact of MLP birth on asthma prevalence and lung function during adolescence remains unclear.

The premature developing brain is exposed to an extrauterine environment during their development. Prematurity is associated to higher risk of cognitive, motor, behavioral and neurosensory deficit. In particular greater prevalence of behavioral and psychiatric disorders have been described in MLP patients (17, 18).

In all these studies, it remains unclear whether the outcomes are associated with gestational age and whether they persist into adulthood. The long-term evolution of MLP adolescents needs to be well characterized to implement specific guidelines, targeted screening, and early treatment to improve their prognosis.

The aim of this study was to characterize the clinical, respiratory (asthma evolution, skin prick test and lung function), cardio-metabolic (hypertension, morphological or functional cardiac changes, growth disturbance and metabolic disorders) and neurodevelopmental outcomes (behavioral, social and learning diseases) among adolescents born with moderate and late prematurity, in comparison to their full-term counterparts.

## Methods

An observational analytic cross-sectional study was performed. All adolescents aged 12 to 15 years, with a history of MLP birth (32–36^6^ weeks of gestational age), born from 1 January 2006 to 31 December 2007, in the Severo Ochoa University Hospital, were invited to participate in the study. A control group of adolescents aged 12 to 15 years born at term (≥37 weeks) was also included. Furthermore, moderate preterm adolescents (MP) (32–33^6^ weeks of gestational age) were compared with late preterm and full-term adolescents (LPFT) (34–41^6^ weeks of gestational age).

The study sample was obtained from the birth registry of the Severo Ochoa University Hospital. A list was generated with all the MLP and full-term infants, arranged in chronological order according to date of birth. All the parents of the MLP patient were contacted by telephone to inform them of the study and, if interested in participate, to arrange an appointment. Each MLP patient was matched with a full-term control, the one immediately after the MLP patient who accepted to be included in the study.

The study was approved by the Ethics Committee of the Severo Ochoa Hospital. Written informed consent was obtained from all the patients and their parents after full explanation of the study protocol. All methods were carried out in the accordance with relevance guidelines and regulations.

Perinatal and neonatal medical history were collected from medical history (newborn measurements were presented with Fenton z-score) (19). The same respiratory, cardiologic, metabolic and neurological evaluation was conducted in both, cases and controls, as described below.

The frequency of asthma, allergy, abnormal lung function, hypertension, functional cardiac changes, growth disturbance, metabolic disorders and behavioral, social and learning diseases was compared between MLP and full-term adolescents and between MP and LPFT adolescents.

### Respiratory evaluation

A specific questionnaire was used to obtain information on wheezing episodes, hospital admissions, maintenance medication, and family history of respiratory disease. The International Study of Asthma and Allergies in Childhood (ISAAC) questionnaire for asthma symptoms for adolescents 13–14 years (20), previously validated and translated to Spanish, was answered by adolescents. *Current asthma* prevalence was estimated by the percentage of children with an affirmative answer to question number 2 (*wheezing or whistling in the chest in the past 12 months*), which has demonstrated the higher correlation with current asthma prevalence in validation studies (20, 21).

Skin prick test was performed to evaluate allergic sensitization for common inhaled allergens. Standardized Allergens (ALK-Abelló) were used with a positive control (10 mg/mL histamine) and negative control (glycerol-saline carrier solution). Positive test was considered when the papule diameter was greater than the positive control (22).

Lung function was evaluated by spirometry, according with the established guidelines (23) using Easy on-PC spirometer (NDD, New diagnostic design medical technologies). At least three reproducible maneuvers were performed, selecting the one with best FEV-1 and FVC values. The percentages were given with Zapletal (24) and z-score of predicted values with reference values of *Global Lung Function Initiative* (25). The variables collected were: FVC, FEV_1_, FEV_1_/FVC and FEF_25–75_. The results were normal when FEV_1_ and FVC were ≥80%, ratio FEV_1_/FVC > 80% and FEF_25–75_ ≥ 65% (26). Bronchodilator test was positive when FEV_1_ increased >12% compared to the baseline after administration of 400 mcg of salbutamol (27).

### Cardiologic evaluation

Blood pressure (BP) was measured oscillometrically with EarlyVue VS30 (Philips Healthcare, EEUU). Results were presented with z-scores of National High Blood Pressure Education Working Group on High Blood Pressure in Children and Adolescents 2004 (28), being considered pathological if >95th percentile (29). Echocardiogram to evaluate the cardiac function was performed using Vivid Pro7 and 9 (General Electric Healthcare, United States). Measurements were obtained using M-mode, power-doppler, continuous-doppler and myocardial performance index (MPI) and the results were presented with z-scores (30–32).

### Metabolic assessment

The follow-up included current anthropometric measurements [weight, height, body mass index presented with Carrascosa 2010 z-score (33)], Waterlow index (34) and abdominal perimeter [presented with Moreno z-score (35)]. Body mass index and abdominal perimeter were considered pathological > 2standard deviations-SD (36, 37). Laboratory tests with metabolic profile (LDL-low-density lipoprotein, HDL-high-density lipoprotein, cholesterol, triglycerides, glycated hemoglobin-HbA1c) were performed. Cholesterol values ≥ 200 mg/dL, HDL < 40 mg/dL, LDL ≥ 130 mg/dL, triglycerides ≥ 150 mg/dL and HbA1c ≥ 6.5% were considered as pathologic (37, 38).

### Neurologic evaluation

Learning disabilities and social development were evaluated. Behavioral tests [*Asperger Syndrome Screening Questionnaire*–ASSQ (39) and attention deficit hyperactivity disorder-ADHD assessment scale (40)] were carried out. The ASSQ was considered abnormal when the score was greater than 19 points. The test ADHD was pathological if the patient obtained >30 points, >10 points in attention deficit/hyperactivity subscale or 11 points in the behavioral disorder subscale (41, 42).

### Statistical analysis

To calculate the sample size, the expected prevalence of asthma in preterm adolescents was expected to be about 25–30% (43) vs. 10% (44) in the control group. The minimal sample size required, with an alpha error of 5% and a power of 80%, was 90 patients in each group. All the analysis was performed using the Statistical Package for the Social Sciences (SPSS) Version 23.0.

Absolute and relative frequencies were used to describe qualitative variables. Continuous variables were described using median and interquartile range-IQR (non-normal distribution). Comparisons were performed with Student’s test, Chi^2^ and Mann–Whitney test. *p*-value < 0.05 was regarded as statistically significant. To control for potentially confounding variables and to examine the independent contribution of the explicative variables on the likelihood of developing asthma, a backward stepwise binomial logistic regression model was built. All the variables with *p*-value < 0.1 were introduced in the multi-variate analysis. Adjusted odds ratios (OR) with 95% confidence intervals (CI) were calculated.

## Results

A total of 150 children (75 preterm and 75 full-term) were included, with mean age 13 years (IQR 13–14). Preterm adolescents were younger (13 years, IQR 12–13) than the full-term ones (14 years, IQR 13–14) (*p* < 0.001). Perinatal characteristics are presented in Table 1.

**Table 1:** Perinatal characteristics of moderate-to-late preterm adolescents vs. the full-term group.

	Moderate-to-late preterm	Full-term	*p*-value	Odds ratio 95% (confidence interval)
	*N* = 75	*N* = 75		
Male gender	36 (48%)	41 (54.7%)	0.414	0.8 (0.6–1.2)
Multiple pregnancy	33 (44%)	2 (2.7%)	**<0.001**	2.6 (2–3.3)
Intrauterine growth restriction	2 (2.7%)	0	0.497	Not available
Preeclampsia	11 (14.7%)	2 (2.7%)	**0.009**	1.8 (1.3–2.4)
Chorioamnionitis	2 (2.7%)	1 (1.3%)	1	1.3 (0.6–3)
Gestational diabetes	9 (12%)	4 (5.3%)	0.245	1.4 (0.9–2.1)
Maternal smoking in pregnancy	9 (12%)	18 (24%)	**0.056**	1.6 (1–2.8)
Maternal age^**^	32 (IQR 31–35)	32 (IQR 29–35)	0.395	
Lung maturation	20 (26.7%)	0	**0.001**	Not available
Gestational age (weeks)^**^	35 (IQR 34–36)	39 (IQR 38–40)	**<0.001**	
Newborn weight (g)^**^	2,300 (IQR 2,090–2,650)	3,280 (IQR 3,040–3,600)	**<0.001**	
Newborn weight (z-score Fenton 2013)^**^	−0.4 [IQR (−0.8) − 0.3]	−0.3 [IQR (−0.8) − 0.2]	0.864	
Neonatal resuscitation	35 (46.7%)	10 (13.3%)	**<0.001**	2.0 (1.5–2.7)
Noninvasive mechanical ventilation	15 (20%)	0	**<0.001**	Not available
Invasive mechanical ventilation	2 (2.7%)	0	0.497	Not available
Patient ductus arteriosus	4 (5.3%)	0	0.120	Not available
Sepsis	10 (13.3%)	2 (2.7%)	**0.031**	1.7 (1.3–2.4)
Hyperbilirubinemia with phototherapy	39 (52%)	0	**<0.001**	Not available
Hypoglycemia	16 (21.3%)	1 (1.3%)	**<0.001**	2.1 (1.6–2.6)

^**^Median (interquartile range-IQR). Values in bold are statistically significant.

### Respiratory health

The responses to the ISAAC questionnaire for asthma symptoms are displayed in Table 2. *Current asthma* (Question 2) was more frequent in the MLP group compared to the full-term one [*p* = 0.008, OR 3 (95% CI 1.26–7.14)] as well as in the MP group in comparison with the LPFT group [*p* = 0.003; OR 3.22 (95% CI 1.56–6.62)]. The frequency of *asthma ever* (Question 6) showed a tendency to be more common in the MLP group, although it did not reach statistical significance (*p* = 0.08).

**Table 2:** Affirmative answers to the ISAAC Questionnaire for asthma symptoms in moderate-to-late preterm vs. full-term adolescents and in moderate preterm vs. late preterm and full-term at the follow up (12–15 years of age).

Answer	Moderate and late preterm	Full-term	*p*-value	Odds ratio 95% (confidence interval)
	*N* = 75	*N* = 75		
1. Have you ever had wheezing episodes at any time?	52 (69.3%)	48 (64%)	0.488	1 (0.8–1.3)
2. Have you had wheezing episodes in the last 12 months?	18 (24%)	6 (8%)	**0.008**	3.0 (1.2–7.1)
3. How many wheezing episodes have you had in the last 12 months? None 1–3 4–12 >12	57 (76%) 14 (18.6%) 2 (2.7%)y2 (2.7%)	69 (92%) 2 (2.7%) 3 (4%) 1 (1.3%)	0.095	Not available
4. How many times had you had symptoms in the night in the last 12 months? Never Less than once a week More than once a week	66 (88%) 7 (9.3%) 2 (2.7%)	73 (97.3%) 2 (2.7%) 0	0.627	Not available
5. Wheezing episodes have you interrupted while speaking in the last 12 months?	5 (27.8%)	0	0.28	Not available
6. Have you ever been diagnosed with asthma?	29 (38.7%)	19 (25.3%)	0.08	1.5 (0.9–2.9)
7. Have you had wheezing episodes while practicing sports?	12 (16%)	9 (12%)	0.48	1.3 (0.6–2.9)
8. Have you had dry in the last 12 months?	7 (9.3%)	2 (2.7%)	0.166	3.5 (0.7–16.3)

	Moderate preterm	Late preterm and full-term	*p*-value	Odds ratio 95% (confidence interval)
	*N* = 17	*N* = 133		
1. Have you ever had wheezing episodes at any time?	9 (52.9%)	91 (68.4%)	0.202	0.8 (0.5–1.2)
2. Have you had wheezing episodes in the last 12 months?	7 (41.2%)	17 (12.8%)	**0.003**	3.2 (1.6–6.6)
3. How many wheezing episodes have you had in the last 12 months? None 1–3 4–12 >12	10 (58.8%) 5 (29.4%) 1 (5.9%) 1 (5.9%)	116 (87.2%) 11 (8.3%) 4 (3%) 2 (1.5%)	0.877	Not available
4. How many times had you had symptoms in the night in the last 12 months?NeverLess than once a weekMore than once a week	13 (76.5%) 4 (23.5%) 0	126 (94.7%) 5 (3.8%) 2 (1.5%)	0.355	Not available
5. Wheezing episodes have you interrupted while speaking in the last 12 months?	5 (27.8%)	0	0.280	Not available
6. Have you ever been diagnosed with asthma?	8 (47.1%)	40 (30.1%)	0.157	1.5 (0.9–2.7)
7. Have you had wheezing episodes while practicing sports?	5 (29.4%)	16 (12%)	0.052	2.4 (1–5.8)
8. Have you had dry in the last 12 months?	3 (17.6%)	6 (4.5%)	0.066	3.9 (1–14.2)

Values in bold are statistically significant.

After logistic regression, *current asthma* and *asthma diagnosis ever* in adolescents were independently associated to neonatal respiratory support and allergic sensitization (Table 3).

**Table 3:** Multivariate analysis of risk factors independently associated with current asthma and asthma diagnosis ever in the whole cohort of 12–15-year adolescents moderate-late preterm and full-term children.

		Adjusted odds ratio	Confidence interval 95%	*p*-value
Current asthma	Neonatal respiratory support	4.7	1.5–15.2	**0.009**
Asthma diagnosis ever		2.9	1.1–7.8	**0.032**
Current asthma	Allergic sensitization	5.7	1.7–18.3	**0.004**
Asthma diagnosis ever		4.8	2.2–10.7	**<0.001**

Values in bold are statistically significant.

Respiratory evolution and family history of asthma/allergic sensitization of preterm and full-term children are shown in Table 4. No differences were observed in asthma chronic treatment prescription or duration of inhaled corticosteroids or anti-leukotrienes treatment. However, MLP patients were more likely to receive longer combined treatment with both drugs simultaneously (*p* = 0.048). The risk of hospital admission due to respiratory causes were similar in both groups.

**Table 4:** Comparison of respiratory evolution during the follow up of moderate-to-late preterm vs. full-term adolescents and in moderate preterm vs. late preterm and full-term at 12–15 years of age.

	Moderate and late preterm	Full-term	*p*-value
	*N* = 75	*N* = 75	
Chronic asthma treatment	23 (30.7%)	24 (32%)	0.860
Duration of anti-leukotrienes treatment (months)^**^	33 (IQR 15–46.5)	36 (IQR 16.5–48)	0.862
Duration of inhaled corticosteroids treatment (months)^**^	24 (IQR 7.5–30)	48 (IQR 12–48)	0.081
Duration of inhaled corticosteroids + antileukotrienes (months) **	36 (IQR 24–60)	24 (IQR 12–42)	**0.048**
Respiratory admissions	27 (36%)	22 (29.3%)	0.384
Respiratory ICU admissions	2 (2.7%)	0	0.155
Allergic sensitization	33 (45%)	34 (45.9%)	0.811
Parental asthma	29 (38.7%)	26 (34.7%)	0.611
Parental atopy	22 (29.3%)	14 (18.7%)	0.126

	Moderate preterm	Late preterm and full-term	*p*-value
	*N* = 17	*N* = 130	
Chronic asthma treatment	7 (41.2%)	40 (30.1%)	0.353
Duration of anti-leukotrienes treatment (months)^**^	0	36 (IQR 15–48)	-
Duration of inhaled corticosteroids treatment (months)^**^	8 (IQR 8)	31.5 (IQR 15–45)	0.308
Duration of inhaled corticosteroids + antileukotrienes (months) **	36 (IQR 21–39)	24 (IQR 12–48)	0.251
Respiratory admissions	7 (41.2%)	42 (31.6%)	0.427
Respiratory ICU admissions	1 (5.8%)	1 (0.7%)	0.214
Passive smoking	3 (17.6%)	70 (52.6%)	**0.009**
Allergic sensitization	9 (52.9%)	58 (43.9%)	0.483
Parental asthma	(4823.5%)	32 (24.1%)	1
Parental atopy	9 (52.9%)	46 (34.6%)	0.139

ICU, Intensive Care Units. ^**^Median (interquartile range-IQR). Values in bold are statistically significant.

Overall, spirometry measurements were within normal limits in both groups. A higher proportion of children with FVC < 80% was observed in the MLP group (*p* = 0.013). Additionally, when comparing MP with LPFT adolescents, FVC < 80% and FEF_25–75_ < 65% were more often found in the MP group (*p* = 0.021 and *p* = 0.046, respectively). Data are represented in Table 5.

**Table 5:** Lung function comparisons between moderate-to-late preterm and full-term counterparts and in moderate preterm vs. late preterm and full-term at the follow up (12–15 years of age).

	Moderate and late preterm	Full-term	*p*-value	Odds ratio (confidence interval 95%)
	*N* = 73	*N* = 74		
FEV_1_ (% predicted)^**^	101.5 (IQR 89.5–110.9)	99.3 (IQR 89.2–107.3)	0.914	
FEV_1_ z-score^**^	0.1 [IQR (−0.9) − 0.9]	−0.1 [IQR (−0.9) − 0.6]	0.955	
FEV_1_ < 80%	7 (9.5%)	3 (4%)	0.209	2.4 (0.6–8.8)
FVC (% predicted)^**^	101.5 (IQR 88.8–108.5)	101.6 (IQR 95.5–109.7)	0.214	
FVC z-score^**^	0.2 [IQR (−0.7) – 0.8]	0.1[IQR (−0.4) – 0.8]	0.547	
FVC < 80%	6 (8.2%)	0	**0.013**	Not available
FEV_1_/FVC (% predicted)^**^	94.1 (IQR 88–105.1)	94.9(IQR 89.8–104.1)	0.077	
FEV_1_/FVC z-score^**^	0.7 [IQR (−1.5) − 0.9]	−0.7 [IQR (−1.4) − 0.6]	0.071	
FEV_1_/FVC ≤ 80%	3 (4.1%)	4 (5.4%)	1	0.8 (0.2–3.2)
FEF_25–75_ (% predicted)^**^	98.4 (IQR 85.8–119.2)	93.9 (IQR 77.5–112.6)	0.341	
FEF_25–75_ z-score^**^	−0.5 [IQR (8–1.6) – 1.1]	−0.3 [IQR (−1.3) − 0.4]	0.579	
FEF_25–75_ < 65%	7 (9.5%)	6 (8.1%)	0.78	1.2 (0.4–3.3)
Bronchodilator test positive	3 (4.1%)	4 (5.4%)	0.628	0.7 (0.2–3.2)

	Moderate preterm	Late preterm and full-term	*p*-value	Odds ratio (confidence interval 95%)
	*N* = 17	*N* = 130		
FEV_1_ (% predicted)^**^	90.9 (IQR 83.9–106.8)	101.4 (IQR 90–109.5)	0.133	
FEV_1_ z-score^**^	−0.8 [IQR (−1.4) – 0.4]	0.1 [IQR (−0.8) – 0.8]	**0.050**	
FEV_1_ < 80%	3 (17.6%)	7 (5.4%)	0.093	3.2 (0.9–11.5)
FVC (% predicted)^**^	97.8 (IQR 81.2–108.9)	101.8 (IQR 94.1–109.3)	0.306	
FVC z-score^**^	−0.2 [IQR (−1.6) − 0.8]	0.2 [IQR (−0.5) − 0.8]	0.241	
FVC < 80%	3 (17.6%)	3 (2.3%)	**0.021**	7.6 (1.7–34.9)
FEV_1_/FVC (% predicted)^**^	94.1 (IQR 90–109.2)	99.6 (IQR 93.4–104.3)	0.830	
FEV_1_/FVC z-score^**^	−0.9 [IQR (−1.3) − 1.7]	−0.1 [IQR (−0.9) – 0.7]	0.767	
FEV_1_/FVC ≤ 80%	2 (11.8%)	5 (3.8%)	0.187	3.0 (0.6–14.5)
FEF_25–75_ (% predicted)^**^	87.6 (IQR 69.5–95.4)	98 (IQR 80.7–114.9)	0.089	
FEF_25–75_ z-score^**^	−0.6 [IQR (−1.5 – 0.2)]	−0.1 [IQR (−0.9) − 0.7]	**0.070**	
FEF_25–75_ < 65%	4 (23.5%)	9 (6.9%)	**0.046**	3.4 (1.2–9.8)
Bronchodilator test positive	0	7 (5.4%)	1	Not available

FEV 1: Forced expiratory volume in one second; FVC: Forced vital capacity; FEF 25-75: Mean forced expiratory flow between 25-75% of FVC. Predicted values presented with Zapletal; Z-score values presented with Global Lung Initiative. Values in bold are statistically significant. **Median (interquartile range-IQR).

After bivariate analysis, the variables associated with lower lung function (FEV_1_ z-score, FEV_1_ < 80%, FVC z-score and FVC < 80%) were: MLP, male gender, *current asthma, asthma diagnosis ever*, wheezing with exercises and chronic asthma treatment (Table 6).

**Table 6:** Variables associated to lower lung function values in moderate-to-late preterm and full-term adolescents (12–15 years of age).

		*p*-value	Odds ratio	Confidence interval 95%
FEV_1_ z-score	Male gender	0.062		
	Current asthma	0.002		
	Wheezing with exercises	0.030		
	Chronic asthma treatment	0.014		
FEV_1_ < 80%	Current asthma	0.001	7.7	2.3–25.2
	Asthma diagnosis ever	0.012	5.0	1.3–18.3
	Wheezing with exercises	0.006	6.0	1.9–18.9
FVC z-score	Male gender	0.011		
	Current asthma	0.023		
FVC < 80%	MPL	0.013	Not available	
	Asthma diagnosis ever	0.073	4.2	0.9–22.4
	Chronic asthma treatment	0.077	4.4	0.8–23.1

FEV 1: Forced expiratory volume in one second; FVC: Forced vital capacity.

### Cardiometabolic results

BP values were similar in MLP and full-term adolescents, with no differences in the prevalence of hypertension. In relation to metabolic disease as dyslipidemia or diabetes, lower HDL values were found only in one MLP and in 4 full-term adolescents. Hypertriglyceridemia was detected in one full-term patient and hypercholesterolemia and high LDL values in only one MLP adolescent. No child presented pathological HbA1c hemoglobin values or metabolic disease (Table 7).

**Table 7:** Blood pressure and metabolic disease in moderate-to-late preterm vs. full-term adolescents and in moderate preterm vs. late preterm and full-term at the follow up (12–15 years of age).

	Moderate and late preterm	Full-term	*p-*value
	*N* = 75	*N* = 75	
Systolic blood pressure^**^	113 (IQR 105–118)	115 (IQR 108–121)	0.064
Systolic blood pressure z-score^**^	0.4 [IQR (−0.3) − 1]	0.4 [IQR (−0.2) − 1]	0.434
Diastolic blood pressure^**^	68 (IQR 64–74)	68 (IQR 66–72)	0.614
Diastolic blood pressure z-score^**^	0.7 (IQR 0.1–1)	0.6 (IQR 0.2–0.8)	0.426
Hypertension (SBP or DBP ≥ p 95)	3 (4%)	4 (5.3%)	1
Cholesterol ≥ 200 mg/dL	1 (1.3%)	0	1
HDL < 40 mg/dL	1 (1.3%)	4 (5.3%)	0.367
LDL ≥ 130 mg/dL	1 (1.3%)	0	1
Triglycerides ≥ 150 mg/dL	0	1 (1.3%)	1
HbA 1c ≥ 6.5%	0	0	Not available

	Moderate preterm	Late preterm and full-term	*p*-value
	*N* = 17	*N* = 133	
Systolic blood pressure^**^	112 (IQR 104.5–119)	115 (IQR 107–120)	0.460
Systolic blood pressure z-score^**^	0.2 [IQR (−0.3) − 1]	0.4 [IQR (−0.2) − 1]	0.654
Diastolic blood pressure^**^	68 (IQR 63–76.5)	68 (IQR 65–72)	0.609
Diastolic blood pressure z-score^**^	0.4 [IQR (−0.1) − 1]	0.4 (IQR 0.1–0.8)	0.868
Hypertension (SBP or DBP ≥ p 95)	1 (5.9%)	6 (4.5%)	0.577
Cholesterol ≥ 200 mg/dL	1 (1.3%)	0	1
HDL < 40 mg/dL	1 (1.3%)	4 (5.3%)	0.367
LDL ≥ 130 mg/dL	1 (1.3%)	0	1
Triglycerides ≥ 150 mg/dL	0	1 (1.3%)	1
HbA 1c ≥ 6.5%	0	0	Not available

High Blood Pressure z-scores presented with scores of National High Blood Pressure Education Working Group on High Blood Pressure in Children and Adolescents 2004. LDL, low-density lipoprotein; HDL, high-density lipoprotein; HbA1c, glycated hemoglobin. ^**^Median (interquartile range-IQR).

Right ventricular function and morphology data are showed in Table 8. MLP adolescents had better TAPSE (*p* = 0.025), TAPSE z-score (*p* = 0.003), shortening fraction (*p* < 0.001) and E/A z-score (*p* = 0.002) than full-term ones. When MP adolescents were compared with LPFT, a smaller right ventricular diastolic diameter and their z-score were observed (*p* = 0.006 and *p* = 0.046, respectively). However, no differences regarding right ventricular function were observed.

**Table 8:** Right ventricular function and morphology in moderate-to-late preterm vs. full-term adolescents and in moderate preterm vs. late preterm and full-term at the follow up (12–15 years of age).

	Moderate and late preterm	Full-term	*p*-value
	*N* = 75	*N* = 75	
Basal diameter (mm)^**^	19.3 (IQR 17.9–21.4)	19.9 (IQR 18.3–22)	0.239
z-score basal diameter^**^	−0.2 [IQR (8–0.6) − 0.1]	−0.3 [IQR (−0.6) − (−0.1)]	0.601
TAPSE (mm)^**^	23.3 (IQR 21.3–25.3)	22.2 (IQR 21–23.3)	**0.025**
TAPSE z-score^**^	0.7 [IQR (−0.5) − 1.8]	−0.1 [IQR (−0.7) − 0.8]	**0.003**
Shortening fraction (%)^**^	37.8 (IQR 32.7–42.9)	30 (IQR 27–32)	**< 0.001**
S′ wave (cm/s)^**^	14 (IQR 13–16)	14 (IQR 13–15)	0.208
S′ wave z-score^**^	0.1 [IQR (−0.5) − 0.7]	−0.1 [IQR (−0.5) − 0.5]	0.074
E/A ratio^**^	1.9 (IQR 1.7–2.2)	1.8 (IQR 1.6–2.1)	0.199
E/A ratio z-score^**^	0.5 (IQR 0.1–1)	0.1 [IQR (−0.3) − 0.8]	**0.002**
E/E’ ratio^**^	3.2 (IQR 2.7–4)	3.2 (IQR 2.8–3.8)	0.937
E/E’ ratio z-score^**^	−0.2 [IQR (−0.7) − 0.3]	−0.4 [IQR (−0.7) − 0.2]	0.341
MPI^**^	0.3 (IQR 0.3–0.4)	0.3 (IQR 0.3–0.3)	0.763
MPI z-score^**^	−0.7 [IQR (−1.1) – (−0.2)]	−0.7 [IQR (−1) – (−0.3)]	0.957

	Moderate preterm	Late preterm and full-term	*p*-value
	*N* = 17	*N* = 132	
Basal diameter (mm)^**^	18.4 (IQR 17.6–19.4)	19.9 (IQR 18.3–21.8)	**0.006**
z-score basal diameter^**^	−0.6 [IQR (−0.8) − (−0.1)]	−0.2 [IQR (−0.6) − 0.1]	**0.046**
TAPSE (mm)^**^	23.4 (IQR 19.7–26.8)	22.7 (IQR 21–24)	0.429
TAPSE z-score^**^	0.4 [IQR (−1.1) − 2.3]	0.2 [IQR (−0.6) − 1]	0.758
Shortening fraction (%)^**^	31.3 (IQR 28.8–35.7)	32.7 (IQR 29–39.5)	0.423
S′ wave velocity (cm/s)^**^	14 (IQR 13–16)	14 (IQR 13–15)	0.617
S′ wave z-score^**^	0.1 [IQR (−0.5) − 0.8]	0.1 [IQR (−0.1) − 0.9]	0.819
E/A ratio^**^	1.8 (IQR 1.5–2.3)	1.9 (IQR 1.7–2.1)	0.754
E/A ratio z-score^**^	0.4 (IQR 0.1–0.5)	0.4 [IQR (−0.1) − 0.9]	0.819
E/E’ ratio^**^	3.2 (IQR 2.9–4)	3.2 (IQR 2.7–3.9)	0.631
E/E’ ratio z-score^**^	−0.4 [IQR (−0.6) − (−0.4)]	−0.3 [IQR (−0.7) − 0.3]	0.901
MPI^**^	0.3 (IQR 0.3–0.4)	0.3 (IQR 0.3–0.3)	0.120
MPI z-score^**^	−0.4 [IQR (−0.9) − 0.5]	−0.7 [IQR (−1.1) − (−0.2)]	0.085

TAPSE, tricuspid annular plane systolic excursion; MPI, myocardial performance index. ^**^Median (interquartile range-IQR). Values in bold are statistically significant.

Regarding the left ventricular assessment, MLP adolescents had smaller ventricular diastolic diameter compared to full-term children (*p* = 0.04) and lower posterior wall z-score values (*p* = 0.037). They also had better S′ wave z-score (*p* = 0.027), E’ wave z-score (*p* = 0.005), E/A ratio (*p* = 0.003) and higher septal MPI z-score (*p* = 0.025). When comparing MP vs. LPFT, no differences in left ventricular morphology and function were found. Data obtained in the assessment of left ventricle are shown in Table 9.

**Table 9:** Left ventricular morphology and function in moderate-to-late preterm vs. full-term adolescents and in moderate preterm vs. late preterm and full-term at the follow up (12–15 years of age).

	Moderate and late preterm	Full-term	*p*-value
	*N* = 75	*N* = 75	
Left ventricle diastolic diameter (mm)^**^	44.1 (IQR 42.2–46.5)	46 (43.6–48.9)	**0.04**
Left ventricle diastolic diameter z-score^**^	−0.2 [IQR (−0.7) − 0.3]	−0.1 [(−0.6) − 0.3]	0.622
Interventricular septum (mm)^**^	6.7 (IQR 5.8–7.6)	6.7 (6.1–7.6)	0.334
Interventricular septum z-score^**^	-0.3 [IQR (−0.9) − 0.2]	−0.5 [IQR (−0.9) − 0.2]	0.343
Left posterior wall (diastole) (mm)^**^	7 (IQR 6.6–7.8)	7.3 (6.3–7.9)	0.644
Left posterior wall z-score^**^	0.4 [IQR (−0.1) − 0.7]	0.2 [IQR (−0.6) − 0.8]	0.037
Shortening fraction (%)^**^	40 (IQR 37–45)	40 (IQR 37–43)	0.529
Ejection fraction (%)^**^	70 (IQR 68–75)	71 (IQR 67–75)	0.440
S′ wave velocity (cm/s)^**^	13 (IQR 12–14)	13.5 (IQR 12–14)	0.600
S′ wave velocity z-score^**^	0.8 (IQR 0.1–1.1)	0.4 (IQR (−0.4) − 0.9)	**0.027**
E wave velocity (cm/s)^**^	22 (IQR 20–23)	20 (IQR 18–24)	0.054
E wave velocity z-score^**^	0.4 [IQR (−0.2) − 1]	0.1 [IQR (−0.5) − 0.6]	**0.005**
E/A ratio^**^	2 (IQR 1.6–2.6)	1.7 (IQR 1.5–2.2)	**0.002**
E/A ratio z-score^**^	0.1 [IQR (−0.6) − 1]	−0.4 [IQR (−0.9) − 0.3]	**0.003**
E/E’ ratio^**^	4.1 (IQR 3.6–4.5)	4.2 (IQR 3.4–5.1)	0.079
E/E’ ratio z-score^**^	−0.6 [IQR (−1) − (−0.3)]	−0.4 [IQR (−1.1) − 0.2]	0.118
Septal MPI^**^	0.3 (IQR 0.3–0.4)	0.3 (IQR 0.2–0.3)	0.144
Septal MPI z-score^**^	−0.8 [IQR (−1.2) − (−0.2)]	−1 [IQR (−1.5) − (−0.5)]	**0.025**
Lateral MPI^**^	0.2 (IQR 0.2–0.3)	0.2 (IQR 0.2–0.3)	0.258
Lateral MPI z-score^**^	−1.1 [IQR (−1.6) − 0.6]	−1.1 [IQR (−1.4) − 0.6]	0.453

	Moderate preterm	Late preterm and full-term	*p*-value
	*N* = 17	*N* = 133	
Left ventricle diastolic diameter (mm)^**^	43.4 (IQR 41.2–46.2)	45.6 (IQR 42.9–47.9)	0.088
Left ventricle diastolic diameter z-score^**^	−0.1 [IQR (−0.6) − 0.2]	−0.1 [IQR (−0.6) − 0.3]	0.885
Interventricular septum (mm)^**^	7 (IQR 5.5–7.8)	6.7 (IQR 6–7.6)	0.924
Interventricular septum z-score^**^	−0.1 [IQR (−1) − 0.4]	-0.4 [IQR (−0.9) − 0.1]	0.397
Left posterior wall (diastole) (mm)^**^	7 (IQR 6.5–7.7)	7.1 (IQR 6.5–7.8)	0.537
Left posterior wall z-score^**^	0.5 (IQR 0.1–0.8)	0.3 [IQR (−0.3) − 0.7]	0.418
Shortening fraction (%)^**^	44 (IQR 37.5–45)	40 (IQR 37–43)	0.339
Ejection fraction (%)^**^	75 (IQR 67.5–76)	70 (IQR 67–75)	0.317
S′ wave velocity (cm/s)^**^	13 (IQR 11.5–14)	13 (IQR 12–14)	0.644
S′ wave velocity z-score^**^	0.4 [IQR (−0.2) − 0.8]	0.6 (IQR 0.1–1.1)	0.324
E wave velocity (cm/s)^**^	22 (IQR 20–23)	21 (IQR 19–23)	0.206
E wave velocity z-score^**^	0.4 [IQR (−0.1) − 0.8]	0.4 [IQR (−0.4) − 0.7]	0.429
E/A ratio^**^	1.8 (IQR 1.6–2.2)	1.8 (IQR 1.5–2.5)	0.821
E/A ratio z-score^**^	−0.3 [IQR (−0.7) − 0.2]	-0.2 [IQR (−0.8) − 0.8]	0.687
E/E’ ratio^**^	4 (IQR 3.1–4.4)	4.2 (IQR 3.5–4.8)	0.129
E/E’ ratio z-score^**^	−0.7 [IQR (−1.3) − (0.3)]	−0.5 [IQR (−1) − (−0.1)]	0.224
Septal MPI^**^	0.3 (IQR 0.3–0.4)	0.3 (IQR 0.2–0.3)	0.137
Septal MPI z-score^**^	−0.6 [IQR (−1.1) − 0.2]	−0.8 [IQR (−1.4) − (−0.3)]	0.054
Lateral MPI^**^	0.3 (IQR 0.2–0.3)	0.2 (IQR 0.2–0.3)	0.458
Lateral MPI z-score^**^	−1 [IQR (−1.3) − (−0.1)]	−1.1 [IQR (−1.5) − (−0.7)]	0.312

MPI, myocardial performance index. ^**^Median (interquartile range-IQR). Values in bold are statistically significant.

### Anthropometric data

No significant differences could be detected between both groups regarding the anthropometric measurements at birth, according to their gestational age (presented with Fenton 2013 z-score). In the follow-up, MLP adolescents had lower weight (*p* < 0.001) and lower weight z-score (*p* = 0.039) than the full-term ones. They also showed lower body mass index (*p* = 0.002) and lower body mass index z-score (*p* = 0.013) than the control group. No differences were found in height or abdominal circumference between the two groups. The Waterlow weight index was lower in the MLP group (*p* = 0.006) and there was a higher percentage of MLP patients with undernutrition index [*p* = 0.04; OR 1.4 (95% CI 1–1.9)]. These differences were also observed when comparing the MP patients with the group composed of LPFT. MP adolescents also had lower weight (*p* = 0.022), weight z-score (*p* = 0.017), body mass index (*p* = 0.011), body mass index z-score (*p* = 0.011) and lower Waterlow weight index (*p* = 0.039). Anthropometrics data of both group of adolescents are shown in Table 10.

**Table 10:** Comparison of anthropometrics characteristics of moderate-to-late preterm vs. full-term adolescents and in moderate preterm vs. late preterm and full-term at the follow up (12–15 years of age).

	Moderate and late preterm	Full-term	*p*-value	Odds ratio (confidence interval 95%)
	*N* = 75	*N* = 75		
Weight (kg)^**^	51.5 (IQR 43.5–60)	55.5 (IQR 49.8–66.3)	**0.001**	
Weight z-score (SD)^**^	−0.3 [IQR (−0.8) − 0.6]	0.1 [IQR (−0.6) − 1]	**0.039**	
Height (cm)^**^	160 (IQR 153.5–172.5)	162.5 (IQR 160–167.7)	**0.001**	
Height z-score (SD)^**^	0.2 [IQR (−0.6) − 1.6]	0.1 [IQR (−0.4) − 0.6]	0.78	
BMI (kg/m^2^)^**^	19.4 (IQR 17–23)	21.4 (IQR 19–25.6)	**0.002**	
BMI z-score (SD)^**^	−0.4 [IQR (−0.9) − 0.5]	0.1 [IQR (−0.5) − 1.2]	**0.013**	
BMI ≥ 2 SD	5 (6.7%)	4 (5.3%)	1	1.2 (0.4–4.5)
Abdominal circumference (cm)^**^	69 (IQR 64–79.5)	72 (IQR 66–80)	0.224	
Abdominal circumference z-score (SD)^**^	0.1 [IQR (−0.5) − 1.6]	0.4 [IQR (−0.5) − 1.7]	0.645	
Abdominal circumference ≥ 2 SD	17 (22.7%)	17 (22.7%)	1	1.0 (0.5–1.8)
Waterloo Index Weight (%)^**^	94.7 (IQR 83–108.4)	102.1 (IQR 89.4–123.4)	**0.006**	
Waterloo index height (%)^**^	99.8 (IQR 97.3–106.2)	98.9 (IQR 98.4–102.5)	0.883	
Waterloo Index weight < 90%	32 (42.6%)	20 (26.6%)	**0.04**	1.4 (1–1.9)
Waterloo index height < 90%	7 (9.3%)	7 (9.3%)	1	1 (0.4–2.7)

	Moderate preterm	Late preterm and full-term	*p*-value	Odds ratio (confidence interval 95%)
	*N* = 75	*N* = 75		
Weight (kg)^**^	47.2 (IQR 42.9–56.6)	55 (IQR 46.9–65.2)	**0.022**	
Weight z-score (SD)^**^	−0.5 [IQR (−0.9)-(−0.1)]	−0.1 [IQR (−0.6) − 1]	**0.017**	
Height (cm)^**^	162 (IQR 154–168.5)	161.5 [IQR 155.5–166.3]	0.922	
Height z-score (SD)^**^	0.1 [IQR (−0.7) − 0.8]	−0.1 [IQR (−0.6) − 0.7]	0.887	
BMI (kg/m^2^)^**^	18.1 (IQR 17–21)	20.4 (IQR 18–25)	**0.011**	
BMI z-score (SD)^**^	−0.6 [IQR (−1) − (−0.1)]	−0.1 [IQR (−0.7) − 1.1]	**0.011**	
BMI ≥ 2 SD	9 (6.7%)	0	0.598	Not available
Abdominal circumference (cm)^**^	67 (IQR 64.2–73.2)	70 (IQR 65.7–80.2)	0.098	
Abdominal circumference z-score (SD)^**^	−0.1 [IQR (−0.8) − 0.7]	0.3 [IQR (−0.5) − 2]	0.113	
Abdominal circumference ≥ 2 SD	0	34 (25.5%)	**0.013**	Not available
Waterloo index weight (%)^**^	92.3 (IQR 81.7–102.3)	98.7 (IQR 86.5–120.6)	**0.039**	
Waterloo index height (%)^**^	99.8 (IQR 96.3–103.1)	99.4 (IQR 97.3–103.3)	0.87	
Waterloo index weight < 90%	7 (41.1%)	45 (33.8%)	0.055	1.2 (0.6–2.2)
Waterloo index height < 90%	2 (11.7%)	12 (9%)	0.661	1.3 (0.3–5.3)

SD, standard deviation. Weight, height, body mass index presented with Carrascosa 2010 z-score. Waterlow index (20) and abdominal perimeter presented with Moreno z-score. ^**^Median (interquartile range-IQR). Values in bold are statistically significant.

After logistic regression, the variables independently associated with undernutrition at 12–15 years of age (Waterloo index weight < 90%) were: male gender [*p* < 0.001; OR 4.65 (95% CI 2.17–9.98)] and MLP birth [*p* = 0.016; OR 2.5 (95% CI 1.19–5.25)].

### Neurological

Neurodevelopmental outcomes are shown in Table 11. No differences were found in the neurodevelopmental and behavioral tests, although MP infants, in comparison with LPFT ones, reported more social problems (*p* < 0.001).

**Table 11:** Neurodevelopmental outcome of moderate-to-late preterm vs. full-term adolescents and in moderate preterm vs. late preterm and full-term at the follow up (12–15 years of age).

	Moderate and late preterm	Full-term	*p*-value
	*N* = 75	*N* = 75	
Learning disability	20 (26.7%)	22 (29.3%)	0.716
Social development	3 (4%)	0	1
Attention deficit hyperactivity disorder	6 (8%)	2 (2.6%)	0.276
ASSQ < 19 points	0	0	Not available
ADHD > 30 points	3 (4%)	3 (4%)	1
ADHD subtype hyperactivity > 10 points	4 (5.3%)	3 (4%)	0.719
ADHD subtype persistent inattention > 10 points	5 (6.7%)	6 (8%)	0.772
ADHD subtype combination of both > 18 points	2 (2.7%)	4 (5.3%)	0.681
ADHD subtype impulsivity > 11 points	7 (9.3%)	4 (5.3%)	0.366

	Moderate preterm	Late preterm and full-term	*p*-value
	*N* = 17	*N* = 133	
Learning disability	4 (23.5%)	38 (28.6%)	0.780
Social development	3 (17.6%)	0	**<0.001**
Attention deficit hyperactivity disorder	1 (5.9%)	7 (5.3%)	1
ASSQ < 19 points	0	0	Not available
ADHD > 30 points	1 (5.9%)	5 (3.8%)	0.523
ADHD subtype hyperactivity > 10 points	1 (5.9%)	6 (4.5%)	0.581
ADHD subtype persistent inattention > 10 points	2 (11.8%)	9 (6.8%)	0.364
ADHD subtype combination of both > 18 points	2 (11.8%)	4 (3%)	0.140
ADHD subtype impulsivity > 11 points	2 (11.8%)	9 (6.8%)	0.364

ASSQ, Asperger Syndrome Screening Questionnaire; ADHD, attention deficit hyperactivity disorder. Values in bold are statistically significant.

## Discussion

To our knowledge, this is the first study to evaluate the global development of MLP patients in adolescence and to compare it with children of the same age born at term. Our results show that MLP adolescents are not the same as full-term, with some differences, but also with some similarities.

In relation to the respiratory evolution, MLP adolescents, exhibited, in our study, higher prevalence of current asthma, with a threefold increased risk compared to full-term children, particularly at lower gestational ages. Furthermore, the MLP group was more likely to undergo longer combined chronic asthma treatment. In summary, these data might indicate that moderate and late birth should be acknowledged as a risk factor for asthma development, not only in the early years of life, but also in adolescence. Several studies have described higher prevalence of asthma in MLP births especially during childhood (4, 43, 45–47). However, the duration of this increased risk is a matter of controversy. Kotecha et al. (48) reported similar respiratory morbidity, including asthma, in 37 born MLP and 34 born full-term at 13–14 years of age. In contrast, Thunqvist et al. (49) found that female subjects, born moderate to late preterm, reported significantly more respiratory symptoms at both, 8 and 16 years of age, than females born term. Additionally, according to the meta-analysis by Been et al. (50), the strength of the association between preterm birth and wheezing disorders is similar between children aged younger and older 5 years. This heterogeneity in the results may be related to various factors, especially the different gestational age of the included patients and their varied age at the time of follow-up, as well as the diversity in the definition of asthma used by different authors. We must also consider other factors that may affect the development of asthma such as a history of asthma, respiratory syncytial virus infection, genetics or the role of inflammasome as an immunomodulator (51). As previously described, we did not find any differences in allergic sensitization rates based on gestational age (14, 16).

Although the rate of asthma admissions did not differ between the preterm and full-term groups, MLP patients did experience higher rate of intensive care unit admissions than full-term children. Our data, according to other reports (12, 16, 45, 52) suggest that, compared to full-term, MLP infants may experience more severe asthma exacerbations (52, 53).

Regarding asthma treatment, no differences were found when the proportion of patients under chronic treatment was compared (30.7 and 32%). However, the duration of combined inhaled corticosteroid/anti-leukotriene treatment was significantly longer in MLP patients, suggesting, a more severe asthma course among MLP adolescents. Other studies have also described similar percentages of chronic asthma treatment in MLP children, such as Perez-Tarazona et al. (16), (28.4%) or Yaacoby-Bianu et al. (54). In contrast, only 8% of moderate preterm children and 5.7% of late preterm in the cohort of Haataja et al. (45) was prescribed asthma treatment.

In relation to lung function, airway obstruction has been described in MLP births during childhood (14, 55). However, the lung function data during school age are controversial (48, 49, 54, 56) and in adolescence most studies (5, 15, 16, 57) indicate that FVC and FEV_1_ values are comparable to those observed in full-term, with only FEF_25–75_ values slightly lower or at the lower limit of normality. Overall, the results of pulmonary function tests in our study showed no significant differences between MLP and full-term children. However, the MP group showed lower FEV_1_ values when compared with LPFT. Furthermore, the probability of FVC values less than 80% and FEF_25–75_ less than 65% was seven and three times higher, respectively, in the MP group. Similar results were observed in previously reports (15, 16, 57). The presence of normal lung function in late adolescents preterm might be related to the pulmonary plasticity described in this group as age progresses (56). On the other hand, the mild lung function impairment is maintained in the most premature (57, 58) with the MP adolescents having, in our cohort, lower FEV_1_ and FEF_25–75_ z-score with a higher percentage of FVC less than 80% and FEF_25–75_ less than 65%. These data suggest that, albeit mild, late preterm infants maintain a mild obstructive and restrictive pulmonary pattern in adolescence, that deserves ongoing monitoring over time to confirm its progression and to implement preventive pulmonary rehabilitation measures aimed at improving thoracic mobility (59, 60).

In our series, no patient had a diagnosis of bronchopulmonary dysplasia. However, Manti el al (61) did not find significant differences in lung function values between very preterm patients (<32 weeks of gestational age) with or without bronchopulmonary dysplasia, at preschool age.

Prematurity has extensively been described as a cardiovascular risk factor (9, 62–64). In our study, no differences in BP values could be demonstrated between MLP and full-term children, even when analyzing separately MP children as a group of greater vulnerability. Several prior publications (4, 9, 64, 65) have noted minimal differences in BP, particularly among infants under 32 weeks, with even smaller variances observed in late preterm infants, primarily identified through continuous blood pressure monitoring. In addition to gestational age, there may be other factors such as a history of small for gestational age (63), female sex (9, 64) as well as other cardiovascular risk factors (obesity, sedentary lifestyle, metabolic disorders, etc.) and prenatal factors (pre-eclampsia, use of corticosteroids or fetal growth, among others) that may influence the increase in BP levels (63–65).

A reduction in cardiac cavity size, coupled with an increase in mass, diminished stroke volume, and decreased end-diastolic volume, along with observed alterations in myocardial deformation and reduced relaxation, have been noted in the hearts of very premature infants (11, 66, 67). These factors collectively contribute to hypertrophy rather than the hyperplasia seen in the third trimester of gestation. Consequently, these alterations lead to shifts in cardiac morphology and affect both systolic and diastolic functions, particularly in the left ventricle. It is important to note that not all studies have reported the same extent of cardiac remodeling, and variations may depend on factors such as perinatal and postnatal care practices, a history of pulmonary hypertension or obesity (64, 66, 68). Regarding cardiac morphology and function in MLP group, our observations revealed a diminished size of the right ventricle with maintained function, consistent with the findings reported by Lewandoski et al. (66). They similarly observed a reduced right ventricle size paired with a larger ventricular mass, though their results exhibited a more pronounced effect. This variation might be partially attributed to the heightened sensitivity of magnetic resonance in detecting changes in ventricular morphology compared to the ultrasound scan utilized in our study. Additionally, the individuals included by Lewandoski et al. (66) were young adults, potentially exposed to various cardiovascular risk factors not yet present in our adolescent patients. Lastly, it is noteworthy that Lewandoski et al. (66) incorporated, not only preterm patients, but also those classified as small for gestational age, a condition with well-documented negative cardiovascular effects. On the other hand, our results are consistent with those published by Arroyas et al. (57), who detected no differences in the morphological assessment of the right ventricle in MLP group. They included patients of the same age than ours and used the same imaging technique (ultrasound) as in our study.

With respect to right ventricular function, Arroyas et al. (57) found a trend toward lower systolic function and worse diastolic function in very premature infants and in those with bronchopulmonary dysplasia. However, these trends were not evident in our study population.

In the evaluation of left ventricle morphology, we observed slightly smaller size z-score values in MLP adolescents and larger septal and posterior wall z-score values. This aligns with studies on preterm infants, which have described a correlation between ventricular size and gestational age, indicating smaller sizes in the more premature infants (66, 69). Additionally, an increase in septal and posterior wall z-score values was noted, a feature independent of blood pressure (66).

In the assessment of the left ventricle function, we observed better diastolic function data in MLP children compared with term children (E-wave velocity, E/A ratio and their respective z-score values). However, no differences were detected in systolic function values, except for the S-wave ´z-score. Notably, these results were not observed in the MP group. Similarly, global left ventricle function (septal MPI index) exhibited higher z-score values in both MLP and MP adolescents. It is crucial to underscore that despite the statistical significance of these differences, they probably lack clinical relevance, as all patients exhibited figures considered normal for their respective age. Our findings on left ventricle function contrast with previously available publications (11, 57, 66, 69) where poorer systolic and diastolic function data were reported in very preterm children. However, it is essential to consider several factors that distinguish these studies from ours. Firstly, the study population in these previous studies consisted of very preterm infants, with an age range between 18 and 40 years, which is significantly older than our cohort. Also, these patients belonged to an era characterized by neonatal care practices markedly different from those of today (such as the use of mechanical ventilation, surfactation or corticosteroids, prenatal factors that influence cardiac remodeling), as well as their patients had a higher cardiovascular risk, since they exhibited hypercholesterolemia, hypertriglyceridemia and insulin resistance, factors not present in our adolescent patients. Additionally, the cardiological assessments were conducted using magnetic resonance imaging, offering a different and potentially more sensitive approach compared to our study.

From an anthropometric point of view, MLP adolescents had less weight and body mass index, without differences in their height when compared to full-term children. The growth of MLP children has been studied in the different stages of development, showing a slower growth in the neonatal and school period (4, 7), followed by a progressive catch-up. When this catch-up occurs is still uncertain, although our results are similar to the findings of Bergmann et al. (8), who observed a slower growth in their premature infants. As far as we know, there are no available data about the nutritional status of MLP children in adolescence. According to our results MLP adolescents have 1.5 times more undernutrition than their full-term pairs. These alterations in anthropometric data had been previously described in selected MP births, but as mentioned above, they are described here for the first time in MLP group.

Catch-up growth has been associated with higher prevalence of obesity in this group of age and possibly, higher cardiovascular risk in adulthood (9, 70). In our study, both groups, MLP and full-term, presented high percentage of pathological abdominal circumference (71), although the percentage of MLP patients with pathological body mass index (>2SD) was similar to the general population (71). However, it is important to highlight that these children have not yet made the catch-up growth and therefore, the risk of obesity might increase later in life.

In our series, no metabolic alterations were found in the MLP group. There has been much controversy in previous publications on this issue. Some studies have described some alterations in the lipid profile, in glycemic values, with increased insulin resistance, and higher frequency of metabolic syndrome (9, 10). However, these studies included young adults, adolescents or even smoking adults as well as patients with different risk factors such as very premature births. In contrast, other studies with a design similar to ours have also reported no metabolic alterations in preterm infants, aligning with our findings (62–64).

Focusing on the neurodevelopment, behavioral, socioemotional and learning difficulties have been described in preterm compared to term infants at 36 months of life (72). Although significant risk factors related to severe prematurity certainly exist, the comparison between the two groups (term and late preterm) demonstrates a similar development pathway in our study, although a higher percentage of social problems was detected in MLP patients. We did not find significant differences related to learning difficulties or need for school or out-of-school support between both groups. This result contrasts with data from other authors (17, 73, 74) who described lower school performance in MLP infants. However, it is worth it considering that these learning difficulties have been observed mainly at school age, but there have not been many follow-up studies in adolescence. According to our results, Alterman et al. (75) confirm previous studies, finding greater learning difficulties at lower gestational age but in the early stages; however, in adolescence only very preterm children had lower academic performance. On the other hand, cognitive problems detected through intelligence questionnaires or other standardized tests are more precise than those reported by the parents as in our study.

In our cohort, MLP adolescents exhibited a notably low prevalence of issues in social relationships, 4%, in contrast to findings reported in other studies (18, 76, 77), such as the study by Palumbi et al. (18), which reported a prevalence of around 30%. The elevated prevalence rates observed in the study by Palumbi et al. (18) could be attributed to the selective sampling of MLP individuals with neuropsychiatric disorders, along with the utilization of diagnostic tests specifically designed to identify these disorders in the studies conducted by Johnson et al. (76) and Polić et al. (77). However, significant differences in social development were observed in the comparison of MP group, suggesting an increase in problems in social relationship with decreasing gestational age.

According to other publications (78, 79) 8% of our MLP adolescents had scores considered diagnostic of ADHD compared to 3% of terms adolescents. Some publications (78, 80, 81) have indicated a higher frequency at lower gestational age, with a risk up to 1.5 times higher in MLP infants compared to full-term. These data were not confirmed in our sample, probably due to the use of different diagnostic scales between studies and different age of the patients.

Few studies refer to the subtypes of ADHD. Preterm birth has been mainly related to inattention disorders (72, 82, 83) with higher risk detected with more severe neonatal pathology and lower gestational age. However, in our sample of MLP adolescents, no significant increase in attention disorders was observed, nor there was a higher risk at a lower gestational age. It is important to consider that the characteristics of the disorder may vary based on gender, age, or developmental stage assessed during the evaluation, warranting further research for the complete validation of this screening scale (41, 84).

The primary limitation of our study was the sample size, which, although larger than in some other studies, proved insufficient for detecting certain differences, particularly in the domain of cardiovascular assessment and in identifying the most vulnerable subgroup of MPs. Additionally, patient recruitment had to be conducted in two stages due to a temporary interruption caused by the confinement measures implemented during the SARS-CoV-2 pandemic. Furthermore, the study faced limitations associated with the requirement for specific diagnostic tests or more sensitive imaging techniques to detect subtle differences.

In summary, despite all possible limitations, our findings provide an overview of the clinical situation of adolescent MLP and suggest that MLP patients should be regarded as a population at heightened risk of developing pathologies, mainly respiratory, minimal alterations on cardiological assessment and possibly anthropometric changes after growth, which may emerge later in life, potentially resulting in significant morbidity and mortality. Therefore, close follow-up of this majority group of preterm infants from the neonatal period to adulthood could be of interest, especially or those with additional risk factors. Prolonged monitoring of this predominant cohort of preterm infants may allow the identification of subtle alterations compared to term and very preterm infants, facilitating early implementation of interventions to mitigate the risk of future morbidity.

## Data Availability

The raw data supporting the conclusions of this article will be made available by the authors, without undue reservation.
